# How to Differentiate between Resistant and Susceptible Wheat Cultivars for Leaf Rust Fungi Using Antioxidant Enzymes and Histological and Molecular Studies?

**DOI:** 10.3390/cells12222643

**Published:** 2023-11-17

**Authors:** Reda I. Omara, Omar Abdullah Alkhateeb, Ahmed Hassan Abdou, Gabr A. El-Kot, Atef A. Shahin, Heba I. Saad-El-Din, Rady Abdelghany, Wasimah B. AL-Shammari, Muayad Albadrani, Yaser Hafez, Khaled Abdelaal

**Affiliations:** 1 Wheat DiseasesResearch Department, Plant Pathology Research Institute, Agricultural Research Center, Giza 12619, Egypt; redaomara43@gmail.com (R.I.O.);; 2 Department of Agribusiness and Consumer Sciences, College of Agriculture & Food Sciences, King Faisal University, Al-Ahsa 31982, Saudi Arabia; 3 Social Studies Department, College of Arts, King Faisal University, Al-Ahsa 31982, Saudi Arabia; 4 Hotel Studies Department, Faculty of Tourism and Hotels, Mansoura University, Mansoura 35516, Egypt; 5 Plant Pathology Branch, Department of Agricultural Botany, Faculty of Agriculture, Kafrelsheikh University, Kafr El-Sheikh 33516, Egypt; 6 Department of Biology, College of Science, University of Hail, P.O. Box 2440, Hail 55476, Saudi Arabia; 7 Department of Family and Community Medicine, College of Medicine, Taibah University, Al-Madinah Al-Munawara 999088, Saudi Arabia; 8 EPCRS Excellence Center, Plant Pathology and Biotechnology Laboratory, Agricultural Botany Department, Faculty of Agriculture, Kafrelsheikh University, Kafrelsheikh 33516, Egypt; hafezyasser@gmail.com

**Keywords:** wheat leaf rust, *Puccinia triticina*, antioxidant enzymes, electrolyte leakage, reactive oxygen species, histological characteristics

## Abstract

Eight wheat cultivars, Sakha-94, Giza-171, Sids-1, Sids-12, Sids-13, Shandweel-1, Misr-1, and Misr-2, were evaluated for leaf rust at the seedling and adult stages in the 2021 and 2022 seasons. Biochemical, histological, and genetic analyses were performed to determine the link between cultivars that were either sensitive or resistant to the disease. Misr-2 and Giza-171 cultivars had the highest levels of resistance to leaf rust races in 2021 (LTCGT, STSJT, and TTTST) and 2022 (MBGJT, TTTKS, and TTTTT) at the seedling stage. However, at the adult stage, Sakha-94, Giza-171, Misr-1, and Misr-2 cultivars had the highest levels of resistance; consequently, they had the lowest final disease severity and the lowest values of AUDPC. The correlation between the seedling reaction and adult reaction was non-significant, with values of 0.4401 and 0.4793 in the 2021 and 2022 seasons, respectively. Throughout the biochemical, histological, and genetic analyses, it was observed that catalase, peroxidase, and polyphenol oxidase activities significantly increased in the resistant cultivars. The discoloration of superoxide (O_2_-) and hydrogen peroxide (H_2_O_2_) significantly decreased in resistant and moderately resistant wheat cultivars (Sakha-94, Giza-171, Misr-1, and Misr-2); higher hydrogen peroxide (H_2_O_2_) and superoxide (O_2_-) levels were recorded for the susceptible cultivars compared to the resistant cultivars. Molecular markers proved that the Lr50 gene was detected in the resistant cultivars. *Puccinia triticina* infections negatively affected most histological characteristics of flag leaves, especially in susceptible cultivars. The thickness of the blade (µ), the thickness of the upper and lower epidermis (UE and LE), the thickness of mesophyll tissue (MT), and bundle length and width in the midrib were decreased in susceptible cultivars such as Sids-1, Sids-13, and Shandwel-1 compared with resistant cultivars.

## 1. Introduction

There are numerous biotic and abiotic factors that affect many plants [1,2,3], such as rice [4], maize [5], and wheat [6]. Wheat (*Triticum aestivum* L.) is one of the most important crops in Egypt and throughout the world [7]. Since wheat is considered a major cereal food crop for humans, keeping productivity and quality at a high level is highly significant to meet the demands of the population [8]. One of the most important costless practices to enhance wheat yield is the appropriate choice of cultivars [9].

*Puccinia triticina*-caused wheat leaf rust is one of the harmful biotic stressors. It is one of three rusts that are destroying Egyptian wheat cultivars [10]. Determining the appropriate cultivar mainly depends on the area of cultivation and sowing time; when seeding is delayed and the weather is favorable, this disease infects sensitive cultivars in a very serious way. Moreover, yield losses in some cultivars may approach 50%, depending on the wheat genotype resistance level and crop development stage at the time of the initial infection [11]. Considering the genetic makeup of the coupling host and parasite, most commercial Egyptian wheat cultivars have suffered unexpected acute disasters in recent years [12]. Egypt is a member of the leaf rust epidemic region, where wheat yield losses are often as high as 50% [13]. Due to their susceptibility in Egypt’s open fields, several varieties, including Giza-158, Mexipak-69, Super-X, and Chenab-70, were eliminated by this disease. Moreover, several wheat cultivars, such as Giza-139, were removed very soon after developing [14]. Thus, resistant cultivars are a key component of the initial disease management strategies for wheat production. Above all, the seeding of a broad-spectrum area with vulnerable types encourages the growth of a massive leaf rust population, creating a breeding ground for mutation and selection [15]. Therefore, the economical and environmentally safe defense of wheat against leaf rust is dependent upon the growth of resistant wheat cultivars.

By partially reducing O_2_- molecules or transferring energy to them, ROS (reactive oxygen species) molecules continuously appear in the chloroplast. Aerobic respiration will inevitably result in the generation of ROS. Four electrons are exchanged and H_2_O_2_ is released when the terminal oxidases, cytochrome oxidase, and alternative oxidase react with O_2_-. O_2_- has frequently been observed to be the first ROS produced [16]. The occurrence of the defense response is ascribed to the buildup of several enzymes, including peroxidase, polyphenol oxidase, chitinase, and 1,3-glucanase. Many host–pathogen combinations, particularly those involving obligate parasites like powdery mildew and rust infections, show a correlation between the up- or downregulation of antioxidant enzymes and resistance [17]. Furthermore, cell membrane stability (electrolyte leakage) is adversely affected by abiotic stress and biotic stress factors [18]. In addition, thinner mesophyll and phloem tissues, as well as a thinner epidermis, were found in susceptible cultivars when compared to resistant cultivars [19]. The major goal of the current study was to use physiological, biochemical, and histological analyses to differentiate between resistance and susceptibility in wheat cultivars inoculated with *Puccinia triticina*.

## 2. Materials and Methods

### 2.1. Evaluation of Eight Wheat Cultivars at Seedling and Adult Stages of Leaf Rust

At the seedling stage, eight wheat cultivars, Sakha-94, Giza-171, Sids-1, Sids-12, Sids-13, Shandweel-1, Misr-1, and Misr-2 (Table 1), were evaluated against the most virulent and frequent races, such as LTCGT, STSJT, and TTTST in 2021 and MBGJT, TTTKS, and TTTTT in 2022, for the leaf rust pathogen in the greenhouse of the Wheat Diseases Research Department, Plant Pathogen Research Institute, ARC, Giza. Ten seeds of each tested wheat cultivar were planted on 10 November in plastic pots (6 cm in diameter). Each pot was filled with a 1:1 blend of soil and peat (*v*:*v*). The eight studied wheat cultivars’ seedlings were inoculated at seven days old by brushing them with urediniospores from the three races in the 2021 and 2022 seasons under greenhouse conditions (20–25 °C, with 80–90% relative humidity, 16 K Lux light intensity, and a 16 h light/8 h dark photoperiod). The inoculation processes were carried out by using the strategies outlined by Stakman et al. [19]. There was a rust reaction twelve days after planting. Leaf rust data were classified by the infection type, with 0 and 1 being considered resistant and 3 and 4 being susceptible [20].

At the adult stage, eight wheat cultivars, namely, Sakha-94, Giza-171, Sids-1, Sids-12, Sids-13, Shandweel-1, Misr-1, and Misr-2, were evaluated in the field over the 2021 and 2022 growing seasons in the Nubaria location (Beheira governorate) (ElEU: 71ft, N: 30*54.52 and E:029*58.04). A randomized complete block design (RCBD) was used to sow these cultivars in three replicates on the first of December. There were three rows in the experimental unit (3 m long and 30 cm apart, with a 5 g seed rate for each row). Morocco and *Triticum spleta saharences* were planted around the perimeter of the experimental area to act as spreaders of leaf rust. In addition to the natural infection, spreaders were intentionally inoculated using a combination of physiological races (LTCGT, STSJT, and TTTST in 2021, as well as MBGJT, TTTKS, and TTTTT races in 2022) during the late tillering and late elongation stages.

### 2.2. he Laboratory Studies

#### 2.2.1. Histochemical Analysis of Reactive Oxygen Species (ROS)

Superoxide (O_2_-) and hydrogen peroxide (H_2_O_2_) were both distinguished by their respective colors: purple for nitroblue tetrazolium (NBT) and reddish-brown for 3,3-diaminobenzidine (DAB). Wheat leaves were vacuum-infused with 0.1 *w*/*v*% NBT (Sigma-Aldrich, Steinheim, Germany) or 0.1 *w*/*v*% DAB (Fluka, Buchs, Switzerland) in a 10 mM potassium salicylate buffer (pH 7.8). Following 20 min and 2 h of daytime incubation, NBT- and DAB-treated samples were cleaned in 0.15 *w*/*v*% trichloroacetic acid in ethanol–chloroform 4:1 *v*/*v* for 1 day [20]. Cleared samples were rinsed with water and placed in 50% glycerol before analysis. Using nicked eyes or a ChemiImager 4000 digital imaging system (Alpha Innotech Corp.; San Leandro, CA, USA), the discoloration of leaves was measured.

#### 2.2.2. Activities of Antioxidant Enzymes

About 0.5 g of freshly treated wheat leaf material was homogenized at 0–4 °C in 3 mL of 50 mM TRIS buffer (pH 7.8), 1 mM EDTA-Na2, and 7.5% polyvinylpyrrolidone to conduct enzyme assays on the wheat plants under investigation. The total soluble enzyme activities in the supernatant of the homogenates were determined spectrophotometrically after centrifugation (12,000 rpm, 20 min, 4 °C) of the homogenates. Using a UV-160A spectrophotometer, all measurements were made at 25 °C (Shimadzu, Nakagyo-ku, Kyoto 604-8511, Japan). The methods of Aebi [21] were used to determine the CAT activity. Using Malik and Singh’s method [22], the activity of polyphenol oxidase (PPO) was measured. The methods of Hammerschmidt et al. [23] were used to measure the peroxidase (POX) activity directly.

#### 2.2.3. Electrolyte Leakage

Twenty 1 cm square discs from wheat leaves were put into flasks one by one with 25 mL of deionized water apiece (Milli-Q 50, Millipore, Bedford, MA, USA). Flasks were shaken for 20 h at room temperature to encourage electrolyte leakage from damaged tissues. An Acromet AR20 electrical conductivity device was used to take each vial’s first electrical conductivity readings (Fisher Scientific, Chicago, IL, USA). The next step was to cause cell rupture by placing the flasks in a hot water bath (Fisher Isotemp, Indiana, PA, USA) for one hour at 80 °C (176 °F). Once more, the vials were shaken on the Innova 2100 platform for 20 h at 21 °C (70 °F). The final conductivity of each flask was assessed, and the electrolyte leakage, as per Szalai et al. [24], was determined as a percentage for each bud by calculating the ratio of initial conductivity to final conductivity multiplied by 100 M.

#### 2.2.4. Histological Studies

Wheat leaf samples 1 cm in length were collected from the middle of the flag leaf at the age of 60 days in the second growing season. Samples were dehydrated in a typical butyl alcohol series, killed and fixed in F.A.A. solution, washed in 50% ethyl alcohol, and then embedded in paraffin wax (56–58 °C). On a rotary microtome model 820, transverse sections 15 microns thick were cut. These sections were dyed with a safranin–light-green mixture and mounted in Canada balsam [25]. Slides were microscopically and photographically analyzed.

#### 2.2.5. Detection of Lr50 in Eight Wheat Cultivars

In the previous eight wheat cultivars, the Lr50 gene was molecularly detected. Using the techniques described by Dellaporta et al. [26], genomic DNA was isolated from the young leaves of plants that were two weeks old. PCR amplification was performed in a thermocycler (Rocorbett-Research, CG1-96, USA), in which a polymerase chain reaction was carried out in a reaction volume of 2.5 L with 2.5 L of genomic DNA containing 50 ng/L, 1 L of each primer (10 pmol, F and R), and 8 L of MQ H_2_O. Specific primers (F: 5′, AAT AAT GTG GCA GAC AGT CTT GG -3 and R: 5′, CCA AGC CCC AAT CTC TCT CT -3′) were used to check for Lr50 in eight cultivars of wheat (MASWheat-ucdavis.edu). The thermocycling conditions for PCR involved 35 cycles of denaturation at 95 °C for 30 s, annealing at 55 °C for 30 s, and extension at 72 °C for 30 s, each spaced 35 cycles apart. A final extension phase that lasted seven minutes at 72 °C was also performed before being held at 4 °C. Amplified products were electrophoresed at 100 V for one hour. Following electrophoresis, the gel was stained with ethidium bromide. Bands were then seen under UV light and captured on camera using a Syngen UV visualizer (gel documentation system, G: BOX). As a standard marker for molecular weight, we used the Mid-Range DNA Ladder 100 bp-3 kbp linear scale (Jena Bioscience, Jena, Germany).

#### 2.2.6. Statistical Analysis

Statistical analysis was carried out using the analysis of variance for a split-plot design according to Gomez and Gomez [27] using the “MSTATC” computer software program. Duncan’s Multiple-Range Test was used to compare treatment means, as described by Duncan [28].

## 3. Results

### 3.1. Evaluation of Eight Wheat Cultivars at Seedling and Adult Stages after Infection with Leaf Rust

**Figure 1:**
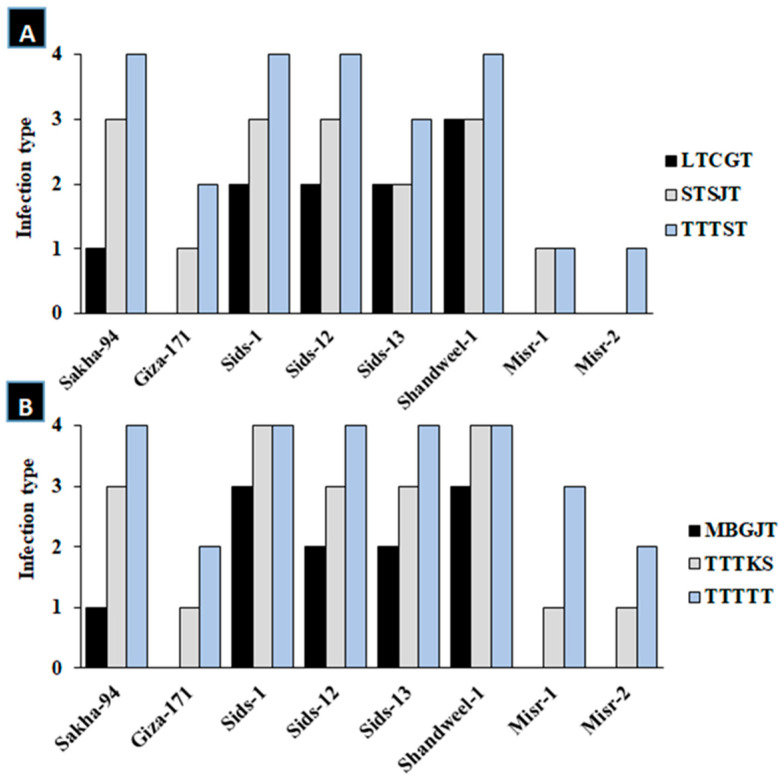
Infection types of eight wheat cultivars at seedling stage with three different races of leaf rust in 2021 (**A**) and 2022 (**B**) seasons.

The data in Figure 1 demonstrate how three races of the leaf rust pathogen, LTCGT, STSJT, and TTTST in 2021, as well as MBGJT, TTTKS, and TTTTT races in 2022, affected the seedling response of eight wheat cultivars at the seedling stage: Sakha-94, Giza-171, Sids-1, Sids-12, Sids-13, Shandweel-1, Misr-1, and Misr-2. Also, according to the data, Misr-1, Misr-2, and Giza-171 in 2021, as well as Giza-171 and Misr-2 in 2022, showed the highest levels of resistance to the leaf rust races, as they displayed low infection types (Figure 1). Whereas LTCGT and MBGJT races were the least aggressive toward the studied wheat genotypes in the 2021 and 2022 seasons, respectively, TTTST and TTTTT races were the most aggressive.

**Figure 2:**
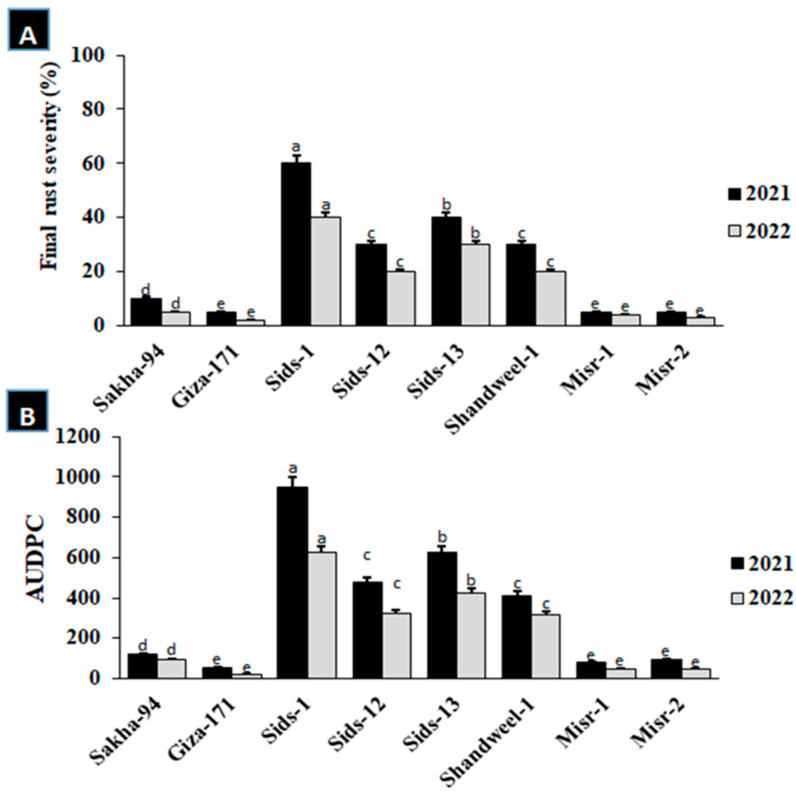
Final disease severity (**A**) and AUDPC (**B**) of eight wheat cultivars at the adult stage after infection with leaf rust in the 2021 and 2022 seasons. (**A**) disease severity, (**B**) AUDPC. Different letters represent significant differences at *p* ≤ 0.05.

At the adult stage, Sakha-94, Giza-171, Sids-1, Sids-12, Sids-13, Shandweel-1, Misr-1, and Misr-2 cultivars were evaluated against leaf rust during the 2021 and 2022 seasons (Figure 2A,B). The resulting disease severity and the values of the area under the disease progress curve (AUDPC) differed between cultivars. The highest final disease severity and values of AUDPC were recorded for Sids-1, Sids-12, Sids-13, and Shandweel-1 during the two seasons (Figure 2A,B). On the other hand, the lowest final disease severity and the lowest values of AUDPC were recorded for Sakha-94, Giza-171, Misr-1, and Misr-2 during the same seasons (Figure 2A,B). According to these findings, cultivars in the first season (2021) exhibited greater final disease severity and AUDPC values compared to the second season (2022), particularly the susceptible cultivars.

### 3.2. Correlation Analysis

**Figure 3:**
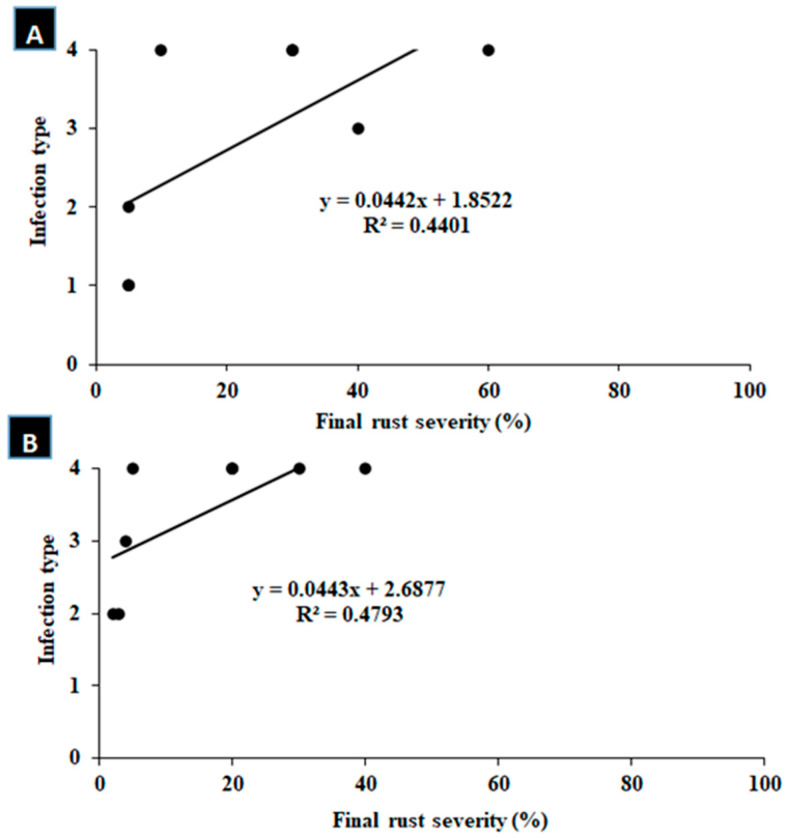
Relationship between infection type at seedling stage and final disease severity at adult stage during 2021 (**A**) and 2022 (**B**) seasons.

Some cultivars had different reactions to the disease in the seedling stage compared to the adult stage: for example, Sakha-94 was susceptible in the seedling stage and resistant in the adult stage. Therefore, the association between the infection type at the seedling stage and disease severity was determined through a correlation analysis over the two growing seasons of the study. Generally, the data presented in Figure 3 indicate a non-significant relationship between them, with values of 0.4401 and 0.4793 in the 2021 and 2022 seasons, respectively.

### 3.3. Activity of Antioxidant Enzymes

**Figure 4:**
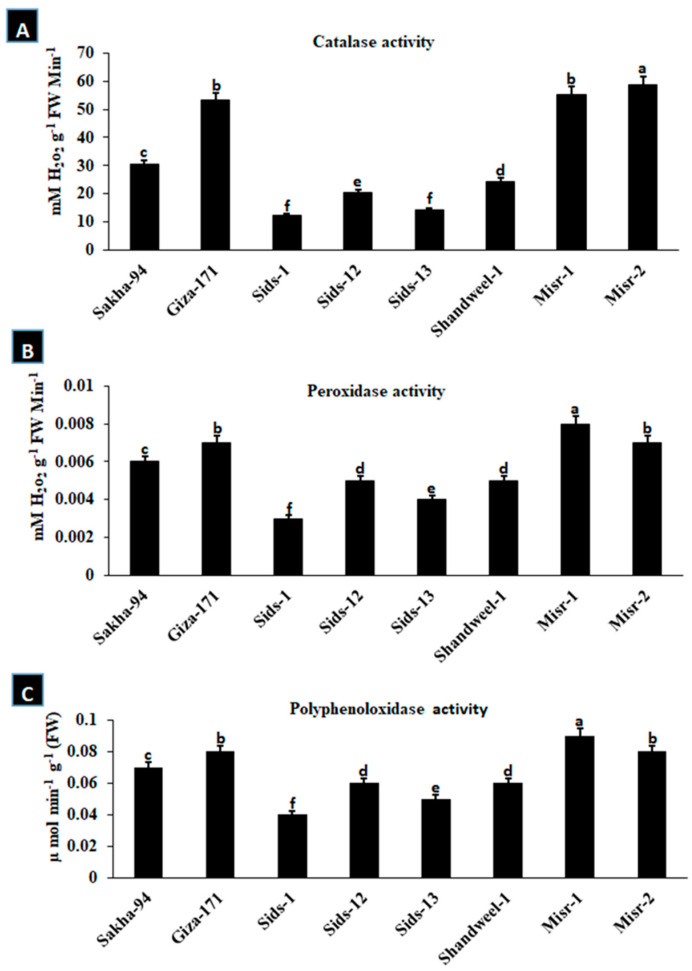
The activities of catalase (**A**), peroxidase (**B**), and polyphenol oxidase (**C**) in some wheat cultivars infected with *Puccinia triticina*. Different letters represent significant differences at *p* ≤ 0.05.

It is well known that the stimulation of ROS-scavenging enzymes is the first reaction after the oxidative burst. Therefore, the enzyme activity, i.e., catalase (CAT), peroxidase (POX), and polyphenol oxidase (PPO), was determined in the wheat cultivars under study. Catalase (CAT), peroxidase (POX), and polyphenol oxidase (PPO) activities were significantly increased in resistant (Sakha-94, Giza-171, Misr-1, and Misr-2) wheat cultivars, as compared to susceptible and moderately susceptible cvs. (Sids-1, Sids-12, Sids-13 and Shandweel-1) (Figure 4A–C).

### 3.4. Electrolyte Leakage

**Figure 5:**
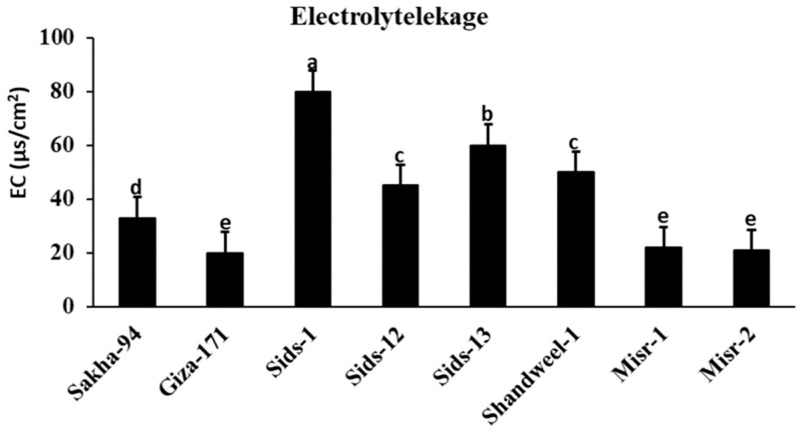
Electrolyte leakage of some wheat cultivars infected with *Puccinia triticina*. Different letters represent significant differences at *p* ≤ 0.05.

Electrolyte leakage (EL), which constitutes an indicator of membrane permeability, was assayed (Figure 5). The infection with *Puccinia triticina* led to a significant increase in electrolyte leakage as an important sign of oxidative stress in susceptible and moderately susceptible wheat cultivars, i.e., Sids-1, Sids-12, Sids-13, and Shandweel-1, compared with the resistant cultivars, i.e., Sakha-94, Giza-171, Misr-1, and Misr-2 (Figure 5).

### 3.5. Histochemical Analysis of Reactive Oxygen Species (ROS)

**Figure 6:**
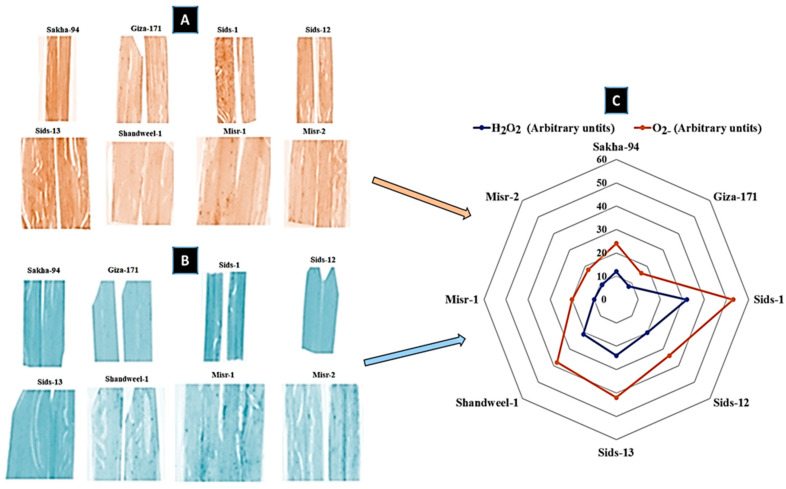
Superoxide and hydrogen peroxide discolorations in some wheat cultivars infected with *Puccinia triticina*. Superoxide (**A**), Hydrogen peroxide (**B**), Comparison (**C**).

Superoxide (O_2_-) and hydrogen peroxide (H_2_O_2_) were visualized as purple and brown discolorations in the eight wheat cultivars (Figure 6). The discoloration significantly decreased in the resistant and moderately resistant wheat cultivars (Sakha-94, Giza-171, Misr-1, and Misr-2) compared with the susceptible cultivars (Sids-1, Sids-12, Sids-13, and Shandweel-1) (Figure 6). These results indicated that the highest hydrogen peroxide (H_2_O_2_) and superoxide (O_2_-) levels were recorded for the susceptible cultivars, Sids-1, Sids-12, Sids-13, and Shandweel-1, compared to the resistant cultivars, Giza-171, Sakha-94, Misr-1, and Misr-2 (Figure 6).

### 3.6. Detection of Lr50 in Eight Wheat Cultivars

**Figure 7:**
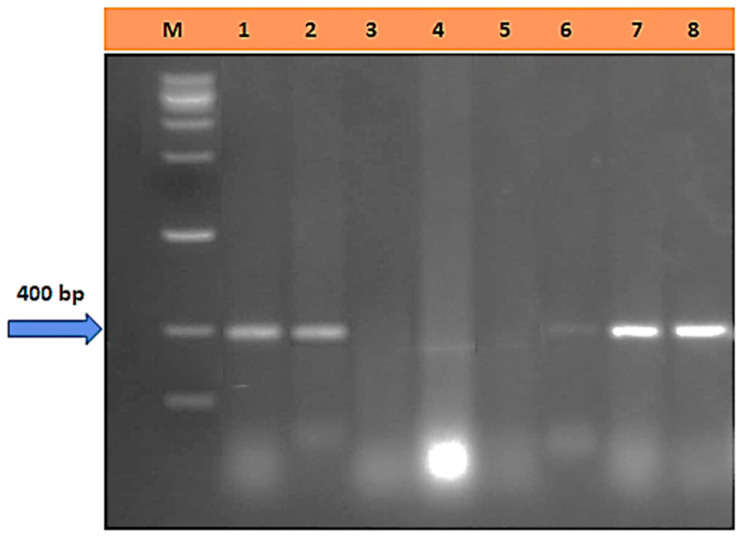
The *Lr50* marker in the amplified DNA was recovered from the 7 cultivars’ electropherogram profiles. M = DNA Ladder (DNA Marker), Lane 1 = Sakha-94, Lane 2 = Giza-171, Lane 3 = Lane 4 = Sids-1, Lane 5 = Sids-12, Lane 6 = Sids-13, Lane 7 = Shandweel-1, Lane 8 = Misr-1 and Lane 9 = Misr-2.

Molecular markers were utilized to identify Lr50 in seven wheat cultivars, including Sakha-94, Giza-171, Misr-1, and Misr-2. Lr50 was detected in Sakha-94, Giza-171, Misr-1, and Misr-2 cultivars at 400 pb and was absent from Sids-1, Sids-12, Sids-13, and Shandweel-1 (Figure 7).

### 3.7. Histological Studies of Eight Wheat Cultivars Infected with P. triticina

**Figure 8:**
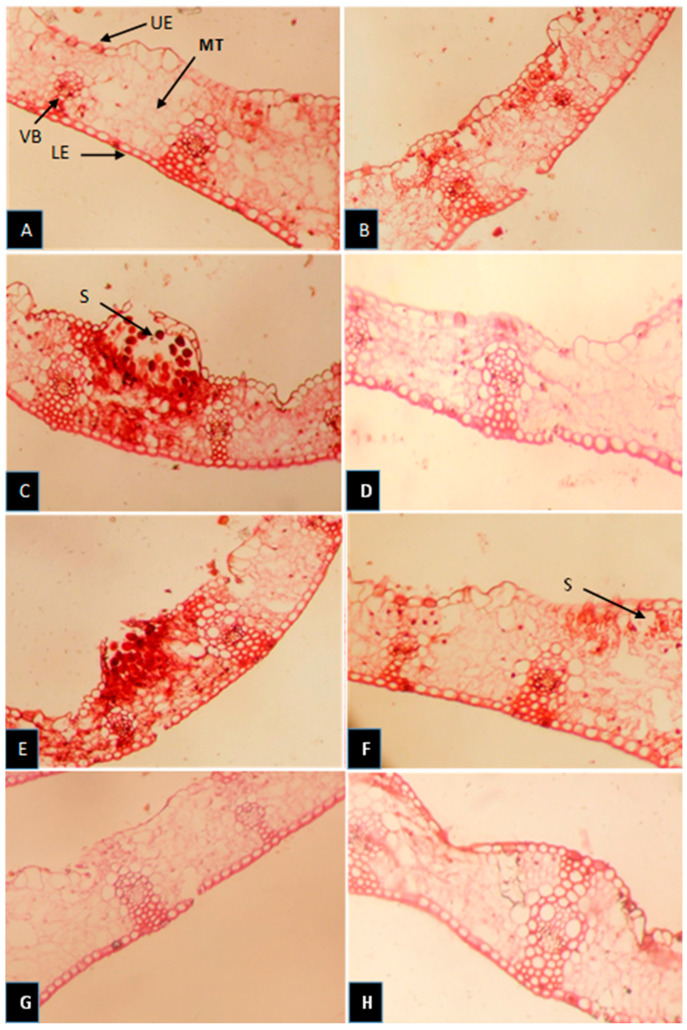
Transverse sections of flag leaves of wheat cultivars ((**A**) Sakha-94; (**B**) Giza-171; (**C**) Sids-1; (**D**) Sids-12; (**E**) Sids-13; (**F**) Shandwel-1; (**G**) Misr-1; (**H**) Misr-2) inoculated with *Puccinia urticaria* during the 2022 growing season (Magnification × 100). Details: UE: upper epidermis; LE: lower epidermis; MT: mesophyll tissue; VB: vascular bundle; S: spores.

The data in Table 2 and Figure 8 show that infection with *P. triticina* negatively affected most histological characteristics of flag leaves, especially in susceptible cultivars. The thickness of the blade (µ), the thickness of the upper and lower epidermis (UE and LE), the thickness of mesophyll tissue (MT), and bundle length and width in the midrib were decreased in susceptible cultivars such as Sids-1, Sids-13, and Shandwel-1 compared with resistant cultivars, i.e., Sakha-94, Giza-171, Misr-1, and Misr-2. Also, there were a large number of spores in the upper epidermis of susceptible cultivars compared with resistant cultivars.

## 4. Discussion

Due to their susceptibility in Egyptian wheat fields, several wheat cultivars, including Giza-139, Mexipak-69, Super-X, and Chenab-70, have been eliminated. In addition, several wheat genotypes, including Giza-139, were abandoned fairly soon after being released [29]. The dynamic population of the causative organism, which generates new virulent races that can overcome their resistance, is mostly to blame for these cultivars’ failure. Hence, throughout the 2021 and 2022 growing seasons, eight cultivars, Sakha-94, Giza-171, Sids-1, Sids-12, Sids-13, Shandweel-1, Misr-1, and Misr-2, were assessed for their resistance to leaf rust at the seedling and adult stages. At the seedling stage, the Giza-171 and Misr-2 cultivars had the highest resistance levels to the most aggressive races, TTTST and TTTTT, in 2021 and 2022, respectively. The cultivars Sakha-94, Giza-171, Misr-1, and Misr-2 had the lowest final disease severity and AUDPC values at the adult stage throughout the course of the two seasons. On the other hand, during the two seasons, Sids-1, Sids-12, Sids-13, and Shandweel-1 had the greatest final disease severity and AUDPC values. These findings generally conflict with those recorded by Abdelbacki et al. [30], who discovered that the cultivars Gemmeiza-11, Sakha-94, Sakha-95, Giza-168, Giza-171, Sids-12, Sids-13, Misr-1, and Misr-2 were resistant to leaf rust during the 2011/2012 and 2012/2013 growing seasons or had only moderate resistance. Moreover, Mabrouk [31] demonstrated that during the growing seasons of 2013–2016, the Gemmeiza-9, Gemmeiza-10, and Gemmeiza-11 wheat cultivars had FRS% ranging from 23.33 to 50% in four locations. These findings could result from the existence of new races in *Puccinia triticina* populations that can disrupt the resistance genes in these cultivars [32]. According to the findings, notably with the susceptible cultivars, the first season (2021) had a higher final disease severity and values of AUDPC than the second season (2022). This is due to yellow rust appearing more aggressively than leaf rust in the second season, in addition to the presence of more aggressive races in this season than in the other. These findings parallel those obtained by Nazim et al. [13], Singh et al. [33], and Boulot [34].

By studying the biochemical alterations, histological features, and genetic makeup of these cultivars, it was possible to analyze the variations in the behavior of the wheat cultivars under investigation as a result of leaf rust. Several studies have demonstrated a connection between the *Puccinia triticina* infection of wheat cultivars and the buildup of reactive oxygen species (ROS), which causes oxidative stress in plants. The continual appearance of ROS in the chloroplast during photosynthesis is caused by the partial degradation of O_2_ molecules or energy transfer to them. Aerobic respiration will inevitably result in the generation of ROS. The most significant ROS linked to oxidative stress are hydrogen peroxide (H_2_O_2_) and superoxide (O_2_), which can activate antioxidant mechanisms even at very low concentrations [35]. In natural circumstances, the overall response to oxidative stress appears to be the upregulation of antioxidant defense systems.

The results of this study revealed that ROS rose in susceptible wheat cultivars like Sids-1, Sids-12, Sids-13, and Shandweel-1 while decreasing in resistant and moderately resistant wheat cultivars like Sakha-94, Giza-171, Misr-1, and Misr-2. The buildup of H_2_O_2_ did not happen in host–pathogen combinations that were sensitive. The antioxidant defense system is known to include enzyme-based antioxidants such as catalase (CAT), peroxidase (POX), and polyphenol oxidase (PPO). According to the study’s findings, catalase (CAT) and peroxidase (POX) activities were significantly higher in resistant wheat cultivars and lower in susceptible ones. These enzymes are crucial when ROS levels are high [35]. Antioxidants are upregulated to protect plant cells from ROS-induced oxidative bursts. This explains why the antioxidant-scavenging enzymes were upregulated in resistant cultivars.

According to recent findings, sensitive wheat cultivars lose more electrolytes than resistant ones. The fact that *P. triticina* is an obligate parasite, highly dependent on its host cells for essential nutrients and chemicals, may be the cause of these results and could explain the significant increase in EL% [36,37]. Host–pathogen compatibility is another factor. This outcome may also result from the pathogen inoculation impacting the vulnerable wheat cultivars’ cell membranes and increasing membrane permeability [38,39,40]. In contrast, infection and a reduction in membrane permeability did not affect the cell membranes of resistant cultivars. These findings concur with those reported by Hafez and Abdelaal [14]. Also, by analyzing the Lr50 gene to differentiate between the cultivars under study, it was found that this resistance gene is present in the resistant cultivars Giza-171, Sakha-94, Misr-1, and Misr-2 and is not present in the susceptible cultivars, i.e., Sids-1, Sids-12, Sids-13, and Shandweel-1 [41]. Most flag-leaf histological characteristics were reduced by *P. triticina* biotic stress, particularly in susceptible cultivars. The epidermal layer, sclerenchyma tissue, and vascular bundles make up the wheat plant’s leaf characteristics [42]. The vascular bundles are arranged in parallel veins and are collateral. It is clear that in susceptible cultivars like Sids-1, Sids-12, Sids-13, and Shandweel-1, histological characteristics, including the blade thickness, upper and lower epidermis thickness, mesophyll tissue thickness, and bundle length and width, were decreased while releasing a large number of spores.

Yet, in susceptible cultivars, xylem vessel diameters were increased. Pereyra et al. [43] also reported that xylem vessel diameters were increased, and the diameter of the vessels plays a crucial role in the adaptation to unfavorable conditions. The cultivars Sakha-94, Giza-171, Misr-1, and Misr-2, which are resistant or moderately resistant, demonstrated an increase in blade thickness, upper and lower epidermis thickness, mesophyll tissue thickness, and bundle length and width. The decrease in the histological characteristics of susceptible cultivars after infection may be due to the fact that *P. triticina* has negative effects on wheat plant growth stages, cell division, and elongation, as well as chlorophyll contents and enzyme activity [44], resulting in a negative effect on the histological characteristics [45].

## 5. Conclusions

At the adult stage, Sakha-94, Giza-171, Misr-1, and Misr-2 cultivars had the highest levels of resistance; consequently, they had the lowest final disease severity and the lowest values of AUDPC. The correlation between the seedling reaction and adult reaction was non-significant, with values of 0.4401 and 0.4793 in the 2021 and 2022 seasons, respectively. Also, catalase, peroxidase, and polyphenol oxidase activities were significantly increased in the resistant cultivars. The discoloration of superoxide and hydrogen peroxide significantly decreased in resistant and moderately resistant wheat cultivars (Sakha-94, Giza-171, Misr-1, and Misr-2). The highest hydrogen peroxide and superoxide levels were recorded for the susceptible cultivars compared to the resistant cultivars. Molecular markers proved that the Lr50 gene was detected in the resistant cultivars. The histological characteristics of flag leaves, especially in susceptible cultivars, were negatively affected. The thickness of the blade (µ), the thickness of the upper and lower epidermis (UE and LE), the thickness of mesophyll tissue (MT), and bundle length and width in the midrib were decreased in susceptible cultivars such as Sids-1, Sids-13, and Shandwel-1 compared with resistant cultivars.

## Figures and Tables

**Table 1:** The pedigrees of eight wheat varieties used in this study.

Variety	Pedigree
Sakha94	OPATA/RAYON//KAUZ CMBW9043180-OTOPM-3Y-010M…
Giza171	Sakha93/Gemmeiza9 S 6-1GZ-2GZ-2GZ-0S
Sids1	HD2172/Pavon“S”//1158.57/Maya74“S”. SD46-4Sd-2SD-1SD-0SD
Sids12	Buc//7c/ald/5/maya74/on//1160-147/3/bb/gll/4/chat“s′”
Sids13	AMAZ19=KAUZ“S”//TSI/SNB“S”
Shandweel1	SITE/MO/4/NAC/TH.AC//3*PVN/3/MIRLO/BUC
Misr1	OASIS/SKAUZ//4*BCN/3/2*PASTOR
Misr2	SKAUZ/BAV92

**Table 2:** Histological characteristics of eight wheat cultivars (flag leaves) infected with *Puccinia triticina*.

Wheat Cultivars	Thickness of Blade (µ)	Thickness of Upper Epidermis (µ)	Thickness of Lower Epidermis (µ)	Thickness of Mesophyll Tissue (µ)	Bundle Length (µ)	Bundle Width (µ)
Sakha-94	217.35	11.81	12.71	124.23	86.72	85.68
Giza-171	220.23	13.23	13.98	128.76	88.76	90.32
Sids-1	202.15	8.54	8.98	104.21	73.74	70.23
Sids-12	210.23	10.98	11.04	112.34	82.47	80.46
Sids-13	208.13	9.87	9.87	110.34	79.23	76.34
Shandweel-1	211.68	10.23	10.65	118.34	81.43	79.68
Misr-1	218.23	13.79	13.22	129.75	89.2	87.22
Misr-2	219.21	13.48	13.79	128.65	86.67	89.34

## Data Availability

Data are contained within the article.
